# Supplementary material for: Microbial biofilm formation and degradation of octocrylene, a UV absorber found in sunscreen
Source: Commun Biol. 2019 Nov 22;2:430. doi: 10.1038/s42003-019-0679-9 (PMC6874559; doi:10.1038/s42003-019-0679-9)
Supplement: Supplementary file 1 — Supplementary Information [file 42003_2019_679_MOESM1_ESM.pdf]

Supplementary Information

Supplementary Tables

Supplementary Table 1

Concentration of octocrylene [mg/L] in the biodegradation assays (Raw data of Figure 3)

**Control (Incubation of octocrylene without *M. agri*)**	**Incubation with *M.agri* and octocrylene**	**Incubation of *M.agri* without octocrylene**
3692	3010	<5
3718	2588	<5
3198	2984	<5
